# Neural cell adhesion molecule regulates chondrocyte hypertrophy in chondrogenic differentiation and experimental osteoarthritis

**DOI:** 10.1002/sctm.19-0190

**Published:** 2019-11-19

**Authors:** Bin‐Feng Cheng, Jun‐Jiang Lian, Hai‐Jie Yang, Lei Wang, Hao‐Heng Yu, Jia‐Jia Bi, Yao‐Xin Gao, Su‐Juan Chen, Mian Wang, Zhi‐Wei Feng

**Affiliations:** ^1^ School of Life Sciences and Technology, Xinxiang Medical University Xinxiang Henan People's Republic of China; ^2^ Shanghai Key Laboratory of Signaling and Disease Research, Laboratory of Receptor‐based Bio‐medicine Collaborative Innovation Center for Brain Science, School of Life Sciences and Technology, Tongji University Shanghai People's Republic of China; ^3^ Henan Key Laboratory of Medical Tissue Regeneration School of Basic Medical Sciences, Xinxiang Medical University Xinxiang Henan People's Republic of China

**Keywords:** chondrocyte differentiation, chondrocyte hypertrophy, mesenchymal stem cells, NCAM, osteoarthritis

## Abstract

Chondrocyte hypertrophy‐like change is an important pathological process of osteoarthritis (OA), but the mechanism remains largely unknown. Neural cell adhesion molecule (NCAM) is highly expressed and involved in the chondrocyte differentiation of mesenchymal stem cells (MSCs). In this study, we found that NCAM deficiency accelerates chondrocyte hypertrophy in articular cartilage and growth plate of OA mice. NCAM deficiency leads to hypertrophic chondrocyte differentiation in both murine MSCs and chondrogenic cells, in which extracellular signal‐regulated kinase (ERK) signaling plays an important role. Moreover, NCAM expression is downregulated in an interleukin‐1β‐stimulated OA cellular model and monosodium iodoacetate‐induced OA rats. Overexpression of NCAM substantially inhibits hypertrophic differentiation in the OA cellular model. In conclusion, NCAM could inhibit hypertrophic chondrocyte differentiation of MSCs by inhibiting ERK signaling and reduce chondrocyte hypertrophy in experimental OA model, suggesting the potential utility of NCAM as a novel therapeutic target for alleviating chondrocyte hypertrophy of OA.

## Graphical abstract

NCAM deficiency enhances hypertrophic chondrocyte differentiation of MSCs by increasing ERK signaling, and accelerates chondrocyte hypertrophy in experimental osteoarthritis.

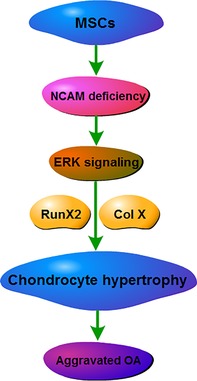

**Significance statement**

Defects in the cartilage are irreversible and difficult to repair in osteoarthritis (OA) patients. A cell‐based therapeutic approach for cartilage regeneration using mesenchymal stem cells (MSCs) has gained attention in recent years; however, chondrogenic differentiation of MSCs is usually inefficient because of excessive chondrocyte hypertrophy‐like change under inflammatory intra‐articular conditions caused by OA. The present study provides the first experimental evidence for neural cell adhesion molecule (NCAM) on the cartilage repair of OA treatment. It has been demonstrated that NCAM deficiency enhances chondrocyte hypertrophy in chondrogenic differentiation of MSCs and in experimental OA, and upregulation of NCAM inhibits hypertrophic chondrocyte differentiation. The results suggest a more efficient strategy for the cartilage repair of OA treatment using NCAM‐overexpressing MSCs.

## 1 INTRODUCTION

Osteoarthritis (OA) is the most common joint disorder worldwide,^1^ showing a progressive increase in the last two decades as the leading cause of large social and economic burden.^2^, ^3^ The study of OA pathophysiology mainly focuses on the mechanisms of cartilage degeneration and chondrocyte biology.^4^ Since chondrocyte is poorly regenerated, osteoarthritic cartilage injury is irreversible and difficult to repair. In recent years, mesenchymal stem cells (MSCs) have become an attractive tool for cartilage regenerative therapy.^5^ MSCs are capable of differentiating into multiple lineages of cells, including chondrocytes.^6^ The process of chondrogenic differentiation of MSCs can be divided into distinct phases including cell condensation, chondrocyte differentiation/proliferation, and chondrocyte hypertrophy.^7^

Chondrocyte hypertrophy is an important physiological process involved in the development of long bones from the cartilage anlagen. This stage is marked by increase in cell volume,^8^ extracellular matrix (ECM) remodeling, and expression of hypertrophic chondrocyte markers such as type X collagen (Col X), matrix metalloproteinase(MMP)‐13, and runt‐related transcription factor 2 (RunX2).^9^ Chondrocyte hypertrophy was shown to be involved in OA pathogenesis. Chondrocytes in healthy cartilage resist proliferation and terminal differentiation whereas chondrocytes in OA resemble hypertrophic differentiation with characteristics of cartilage matrix remodeling, cartilage calcification, and expression of hypertrophy markers including RunX2 and Col X.^10^, ^11^ Understanding the molecular mechanisms regulating chondrocyte hypertrophy is important for clinical MSCs application and drug development to repair cartilage defects of OA.

Neural cell adhesion molecule (NCAM), a member of immunoglobulin superfamily that mediates cell‐cell and matrix interactions,^12^ plays a key role in regulation of neurite outgrowth, synaptic plasticity, neuronal development, learning, and memory.^13^, ^14^, ^15^ NCAM is also expressed in MSCs,^16^, ^17^ but its function remains largely unknown. We have previously demonstrated that NCAM promotes adipocyte differentiation of murine MSCs via PI3K‐Akt pathways.^18^ NCAM is also involved in chondrogenesis. In the process of chondrogenic differentiation, NCAM is expressed in prechondrogenic cells and increased during cell condensation,^19^ but it becomes undetectable in hypertrophic chondrocytes.^20^, ^21^ Previous studies showed that NCAM initiated chondrogenesis by promoting and stabilizing the condensation step while may not contribute to chondrocyte differentiation directly.^22^, ^23^ However, the role of NCAM in chondrocyte hypertrophy is still poorly understood.

In the present work, we evaluated the effects of NCAM on chondrocyte hypertrophy in murine MSCs, in chondrogenic ATDC5 cells, and in experimental murine OA. We also investigated the underlying signaling pathway in MSCs involved in hypertrophic differentiation of chondrocytes.

## 2 MATERIALS AND METHODS

### 2.1 Materials

MEK inhibitor U0126, transforming growth factor β1 (TGF‐β1), ascorbic acid‐2‐phosphate, dexamethasone, and fibroblast growth factor (bFGF) were obtained Sigma‐Aldrich (St. Louis, MO, USA). Fetal bovine serum (FBS) was purchased from Gibco (Grand Island, New York, USA). The mammalian expression vector pcDNA4/Myc was obtained from Invitrogen (Carlsbad, California, USA). siRNA expression vectors pSilencer 4.1‐CMV neo was obtained from Ambion (Austin, Texas, USA). Lipofectamine 2000, G418 and puromycin were purchased from Invitrogen. Other chemicals were purchased from Sigma‐Aldrich. Antibodies against RunX2, phospho‐ERK, ERK2, c‐Myc, and β‐actin were purchased from Cell Signaling Technology Inc (Beverly, Massachusetts, USA). Antibody against NCAM was obtained from Millipore (Billerica, Massachusetts, USA). Antibody against Col X was obtained from Abcam (Cambridge, Massachusetts, USA).

### 2.2 NCAM‐deficient mice and cell culture

The *Ncam* ^−/−^ (knockout; KO) mice were generated on a C57/BL6 background as previously described.^24^ Wild‐type (WT) and KO MSCs were obtained from 8‐week‐old male mice as previously described.^18^ Briefly, cells were harvested from mouse bone marrow and cultured in low glucose Dulbecco's modified Eagle's medium (DMEM‐LG) containing 10% FBS, 100 IU/mL penicillin and 100 g/mL streptomycin. Nonadherent hematopoietic cells were discarded after incubation for 7 days, and the adhered MSCs were purified by repeated passaging. MSCs with fibroblast‐like morphology from passage 6 to 15 were used in this study.

The pre‐chondrocyte cell line, ATDC5, was purchased from the RIKEN Cell Bank (Ibaraki, Japan). Cells were cultured in DMEM/F‐12 medium (Hyclone) containing 5% FBS, penicillin (100 U/ mL), and streptomycin (100 g/mL).

### 2.3 Chondrogenic differentiation

Chondrogenic differentiation of MSCs was conducted by incubation in differentiation medium supplemented with 10 ng/mL TGF‐β1, 50 nM ascorbic acid‐2‐phosphate, 0.1 μM dexamethasone, and 10 ng/mL bFGF. Chondrogenic differentiation of ATDC5 cells was conducted by incubation in differentiation medium supplemented with 10 μg/mL insulin (Sigma‐Aldrich), 5.5 μg/mL transferrin (Sigma‐Aldrich), and 5 ng/mL sodium selenite (Sigma‐Aldrich).

### 2.4 Quantitative real‐time PCR

Total RNA was extracted with TRIzol (Invitrogen) according to the manufacturer's instructions, and cDNA was synthesized using a High‐Capacity cDNA Reverse Transcription Kit (Applied Biosystems, Foster City, California). The mRNA levels were measured using an ABI Prism 7500 Sequence detection system (Applied Biosystems) and SYBR Green qPCR Master Mix (KAPA Biosystems). The expression levels of each gene were normalized using the internal reference gene *gapdh*. The qPCR primers were designed and shown in Table S1.

### 2.5 Western blotting

Cells and tissue samples were lysed with radio immunoprecipitation (RIPA) buffer and lysed on ice supplemented with proteinase and phosphatase inhibitors cocktail (Sigma‐Aldrich). Protein concentrations were determined using the BCA Protein Assay Kit (Pierce Biotechnology Inc, Rockford, Illinois). Equal amounts of protein were separated by sodium dodecyl sulfate‐polyacrylamide gel electrophoresis and transferred to polyvinylidene difluoride (PVDF) membrane. After blocking with 3% bovine serum albumin (BSA; Sigma‐Aldrich) for 1 hour, the membranes were incubated with primary antibodies against NCAM, Col X, RunX2, phospho‐ERK, ERK2, c‐Myc, or β‐actin overnight at 4°C. Subsequently, the membranes were washed three times with TBST and incubated with the secondary antibody for 40 minutes. Membranes were scanned by the ImageQuant LAS 4000 system (GE Healthcare) and images, in some cases, were analyzed using the ImageJ software.

### 2.6 Alizarin Red staining and quantification

Cells were fixed in 4% paraformaldehyde (PFA) for 30 minutes followed by staining with 40 mM Alizarin Red S (ARS; pH 4.1) for 30 minutes. Quantitative analysis of ARS was performed by incubating the stained wells with 10% cetyl pyridinium chloride monohydrate for 30 minutes at room temperature before centrifugation. The extracted supernatant was measured at 562 nm using a Molecular Devices microplate reader.

### 2.7 Immunofluorescence staining

Cells were fixed with 4% paraformaldehyde for 30 minutes and then permeabilized in 0.5% Triton X‐100 (Sigma‐Aldrich) for 30 minutes at room temperature. Permeabilized cells were blocked with 3% BSA for 1 hour at room temperature and then stained overnight with primary antibody against Col X at 4°C. Subsequently, cells were stained with corresponding secondary antibody (Cell Signaling Technology Inc) for 45 minutes at 4°C in the dark and incubated for 30 minutes in dimethylsulfoxide for nuclei visualization. Confocal imaging was carried out using a fluorescence microscope (Leica DMIL LED). Images represent the z‐stack projection of confocal sections.

### 2.8 Induction of experimental OA rats

Wistar rats (male weighing between 180 and 220 g) were purchased from the Experimental Animal Center of the National Institute for the Control of Pharmaceutical and Biological Products (Beijing, China). All animal experiments were approved by the Ethic Committee of Xinxiang Medical University. The rats were anaesthetized intraperitoneally (i.p.) with chloralic hydrate (300 mg/kg). For monosodium iodoacetate (MIA; Sigma‐Aldrich) induced OA (n = 7 per group), rats received intra‐articular knee injections with MIA (20 mg/mL, 30 μL) or sterile saline as a control. The rats were sacrificed on day 21, cartilage samples were obtained for analysis of NCAM by Western blotting. Serum was obtained for detection of interleukin (IL)‐1β and tumor necrosis factor (TNF)‐α using enzyme‐linked immunosorbent assay (ELISA).

### 2.9 Induction of experimental OA mice

The KO and WT mice (male and female, 8‐12 weeks, 20‐30 g, n = 5 per group) were anaesthetized with chloralic hydrate (400 mg/kg, i.p.). OA mice model was induced by an injection of MIA (20 mg/mL, 15 μL) in sterile saline into the left knee joint cavity through the patellar ligament. On day 10 or 21, the whole knee joints were obtained for histological or immunohistochemical study. Serum was obtained at day 21 for detection of IL‐1β and TNF‐α using ELISA. All animal experiments were approved by the Ethics Committee of Xinxiang Medical University.

### 2.10 Histological analysis and immunohistochemical staining

For histological analysis, the whole knee joints were fixed in 4% PFA, decalcified, embedded in paraffin, sectioned, and stained with Safranin O/Fast Green or hematoxylin and eosin (H&E) using standard protocols. For immunohistochemical staining, sections were pretreated with trypsin, incubated with primary antibody against RunX2 at 4°C overnight, then incubated with secondary antibody, and stained using standard protocols.

### 2.11 Plasmid constructs and transfection

siRNA vectors silencing NCAM and plasmids expressing full‐length mouse NCAM were designed and constructed as previously described.^18^ The expression plasmid containing constitutive active form of MEK was a gift from Sheng‐Cai Lin (Xiamen University, China). Transfection was conducted using Lipofectamine 2000 following manufacturer's instructions. To obtain stable mixed cell lines, cells were selected with zeocin at 150 μg/mL or neomycin at 800 μg/mL for 10‐14 days. Gene silencing or overexpression of NCAM was validated by Western blotting with anti‐NCAM or anti‐c‐Myc tag antibodies.

### 2.12 Statistical analysis

All data are presented as mean ± SEM unless otherwise specified. Statistical analysis was determined with GraphPad Prism (GraphPad Software, La Jolla, California). Comparisons between two groups were performed using a two‐tailed unpaired Student's *t* test, comparisons between three or more groups were analyzed using one‐way ANOVA followed by Tukey's post hoc test, Welch correction was used to protect against heteroscedastic data sets. A *P* value <.05 was regarded as statistically significant.

## 3 RESULTS

### 3.1 NCAM deficiency accelerates chondrocyte hypertrophy in experimental OA mice

**Figure 1:**
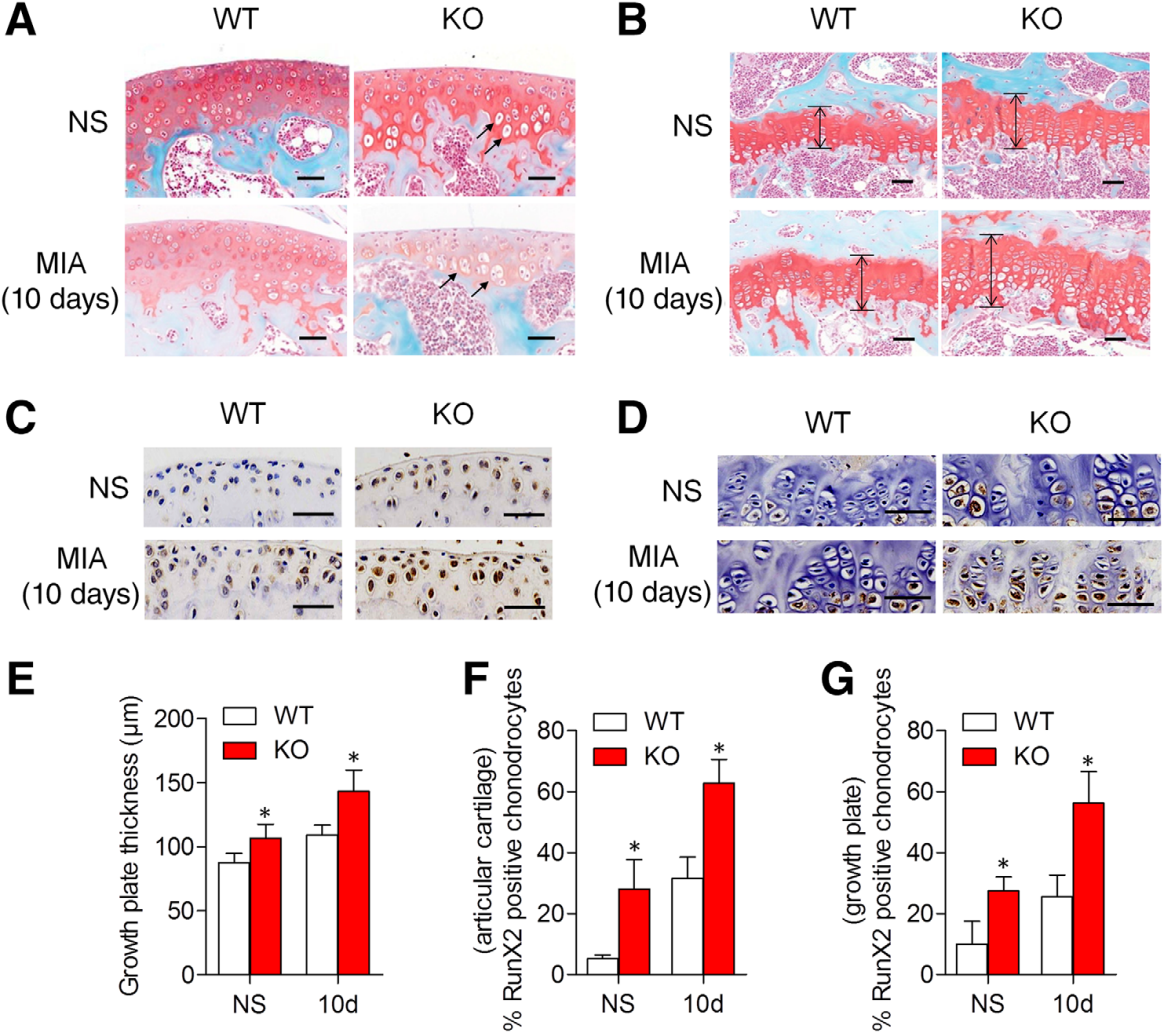
Neural cell adhesion molecule (NCAM) deficiency accelerates chondrocyte hypertrophy in experimental osteoarthritis (OA) mice. Safranin O staining of articular cartilage (A) and growth plate (B) sections in MIA‐induced wild‐type (WT) and *Ncam* ^−/−^ (KO) mice, arrowheads point to hypertrophic chondrocytes (black arrows in A). The normal saline (NS) was used as sham group (n = 5, original magnification ×200, scale bar 50 μm). E, Quantification of growth plate thickness (black arrows in B), mean ± SD, *P < .05, compared with the NS group. Immunohistochemical staining for RunX2 levels of articular cartilage (C) and growth plate (D) in MIA‐induced WT and KO mice (n = 5), images are shown at 200‐fold magnification, scale bar 50 μm. Percentage of RunX2‐stained cells of articular cartilage (F) and growth plate (G) in MIA‐induced WT and KO mice (mean ± SD, *P < .05, compared with the NS group)

To investigate the role of NCAM in chondrogenesis, the *Ncam* ^−/−^ (knockout; KO) mice were generated. Chondrocyte hypertrophy of articular cartilage was observed in *Ncam* ^−/−^ mice of sham group (Figure 1A). Histologic results showed more hypertrophic chondrocytes in growth plate cartilage area in sham *Ncam* ^−/−^ mice as compared to sham WT mice (Figure 1B). The data suggest that NCAM deficiency enhances chondrocyte hypertrophy in chondrogenic differentiation in vivo. Chondrocyte hypertrophy‐like changes play a crucial role in OA cartilage degeneration.^25^ To elucidate the role of NCAM in OA, experimental OA mice were induced by MIA in WT and *Ncam* ^−/−^ mice. Cell clusters, reduced matrix staining, and severe chondrocyte hypertrophy in articular cartilage and growth plate of *Ncam* ^−/−^ mice were notably accelerated, early observed in 10 days (Figure 1A,B), whereas similar degree of destruction emerged in cartilage of WT mice until 21 days (Figure S1). The growth plate thickness was also increased with more hypertrophic chondrocytes (Figure 1E). The hypertrophy‐characteristic marker RunX2, vital for promoting chondrocyte maturation into hypertrophic chondrocytes and drive the expression of the terminal differentiation markers,^26^, ^27^ was detected by immunohistochemistry. In sham mice, the level of RunX2 in articular and growth‐plate cartilage of *Ncam* ^−/−^ mice was higher than that in WT mice (Figure 1C,D). In OA mice, both WT and *Ncam* ^−/−^ mice produced more RunX2 protein. However, the expression of RunX2 is much higher in the cartilages of *Ncam* ^−/−^ mice than that in WT mice (Figure 1F,G). These results indicate that NCAM plays a crucial role in the progression of chondrocyte hypertrophy in chondrogenic differentiation and OA model in vivo.

### 3.2 NCAM deficiency enhances chondrocyte hypertrophy in chondrogenic differentiation of mouse MSCs

**Figure 2:**
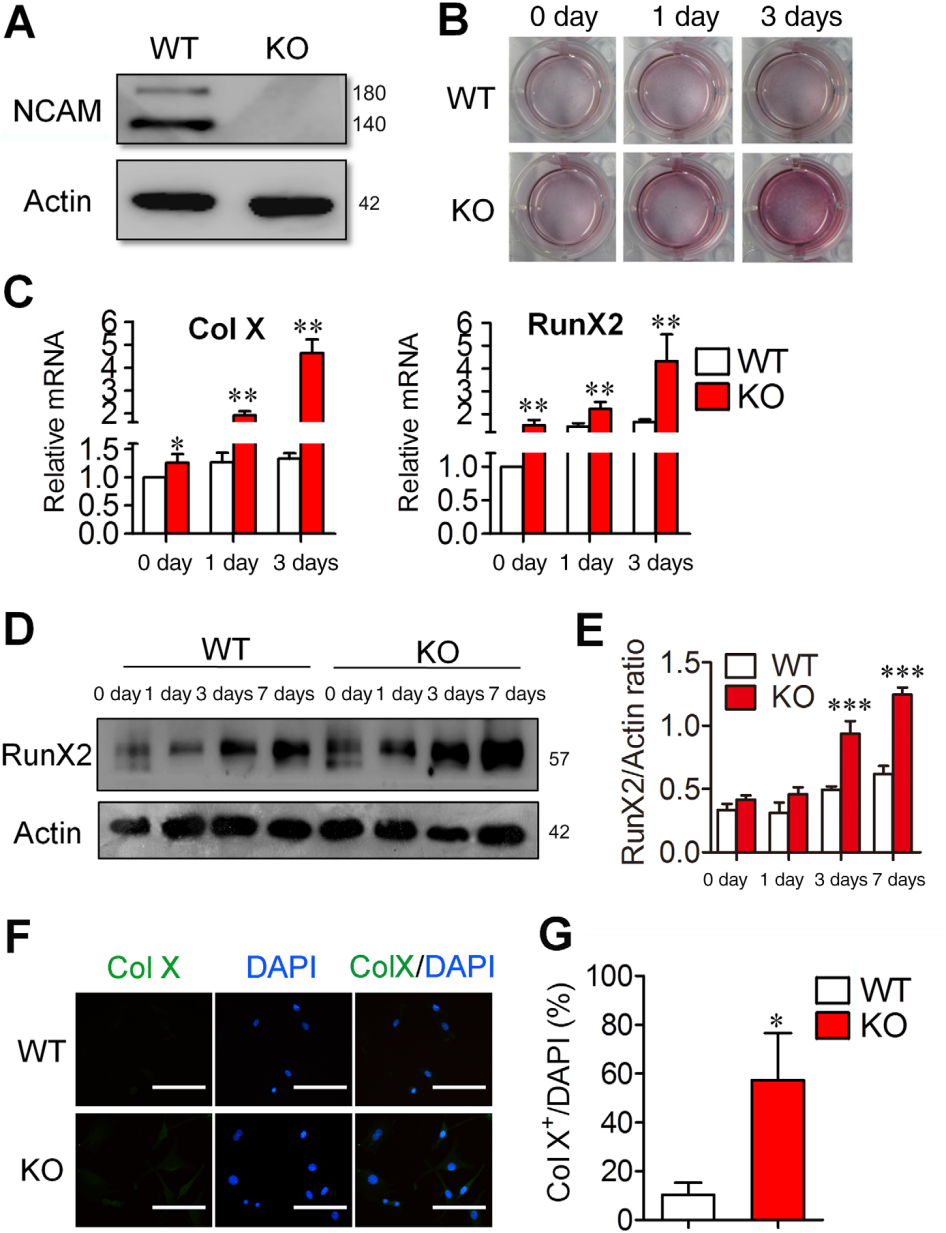
Neural cell adhesion molecule (NCAM) deficiency leads to increased hypertrophic chondrocyte differentiation of mouse mesenchymal stem cells (MSCs). A, Wild‐type (WT) and *Ncam* ^−/−^ (KO) MSCs were examined with anti‐NCAM antibody. β‐actin was detected as a loading control. B, Matrix mineralization of differentiated WT and KO MSCs was assessed by Alizarin red staining. C, The expression of RunX2 and Col X in chondrocyte‐differentiated WT and KO MSCs was analyzed by real‐time PCR. The results are expressed as the mean ± SEM of three independent experiments. *P < .05, **P < .01, compared with differentiated WT MSCs. D, The expression of RunX2 was analyzed by immunoblotting. Cells were induced with chondrogenic media for 0, 1, 3, and 7 days; β‐actin was detected as a loading control. E, Level of RunX2 was quantified by densitometry and normalized to β‐actin. Data are representative of three independent experiments and values are means ± SEM. ***P < .001, compared with WT cells. F, The expression of Col X in chondrocyte‐differentiated WT and KO MSCs was detected with immunofluorescence microscopy (original magnification ×200, scale bars 100 μm). G, Quantification of Col X expression in chondrocyte‐differentiated WT and KO MSCs. Data are representative of three independent experiments and values are means ± SEM. *P < .05, compared with WT cells

To study the role of NCAM in the chondrogenic differentiation of MSCs, we isolated MSCs from WT and *Ncam* ^−/−^ mice. The Western blot results confirmed the expression of NCAM in WT MSCs but not in *Ncam* ^−/−^ cells (Figure 2A). In WT MSCs, proteoglycan and matrix mineralization were observed in 7 and 10 days after chondrogenic differentiation, respectively (Figure S2A). Interestingly, mineralization was markedly increased in *Ncam* ^−/−^ MSCs, as revealed by Alizarin red staining 3 days after chondrogenic differentiation (Figure 2B and Figure S2B), whereas Alcian blue staining was undetectable in both WT and KO cells (Figure S2C). The mRNA expressions of hypertrophic markers *Col 10a1* (Col X) and *RunX2* were upregulated in *Ncam* ^−/−^ MSCs as compared to WT cells (Figure 2C), whereas the mRNA levels of chondrocyte differentiation genes *Sox9* and *Col 2a* (collagen II) were comparable between the two groups (Figure S3). To further confirm the effect of NCAM deficiency on hypertrophic differentiation, the protein levels of hypertrophic marker RunX2 were detected by Western blotting. As shown in Figure 2D,E, the induction of RunX2 in *Ncam* ^−/−^ MSCs was higher than that in WT cells. The immunofluorescence assay was also employed to examine the expression of another hypertrophic marker Col X. Like the expression pattern of RunX2, Col X expression was upregulated in *Ncam* ^−/−^ MSCs (Figure 2F,G). These findings further support the conclusion that loss of NCAM function promotes hypertrophic differentiation during chondrogenic differentiation of MSCs.

### 3.3 NCAM silencing boosts chondrocyte hypertrophy in differentiation of prechondrogenic ATDC5 cells

**Figure 3:**
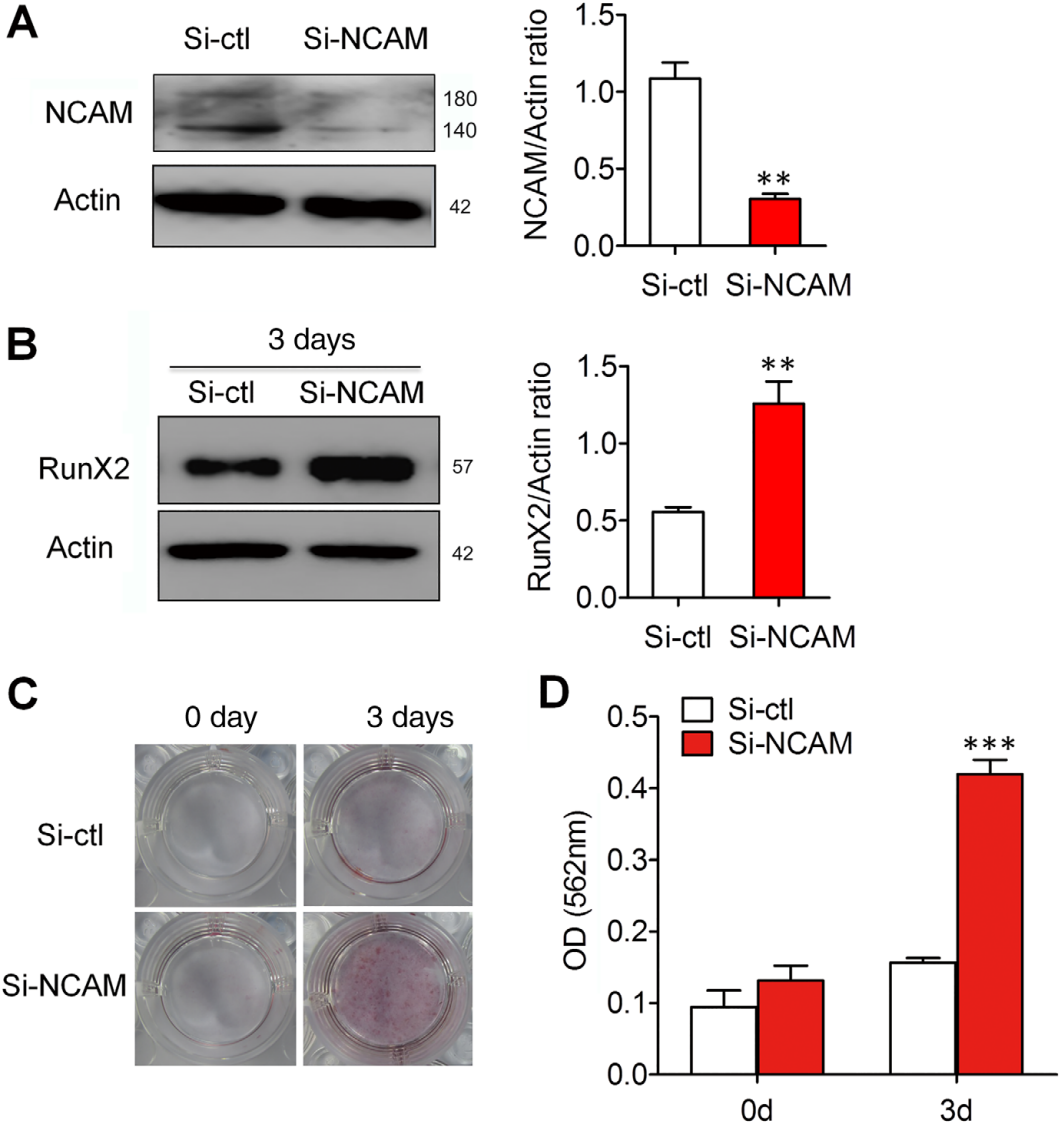
Neural cell adhesion molecule (NCAM) silencing promotes hypertrophic chondrocyte differentiation of prechondrogenic cell line ATDC5. A, Cells were transfected with pSilencer‐4.1‐based plasmid containing a scrambled sequence (control; Si‐ctl) or a 19‐bp oligonucleotide insert targeting mouse NCAM (Si‐NCAM), and analyzed by immunoblotting with anti‐NCAM antibody. Level of NCAM was quantified by densitometry and normalized to β‐actin (n = 3; mean ± SEM; **P < .01, compared with control siRNA group). B, Cells underwent chondrogenic differentiation for 3 days and the expression of RunX2 was analyzed by immunoblotting (n = 3; mean ± SEM; **P < .01, compared with Si‐ctl group). C, Cells transfected with control or NCAM siRNA were induced with chondrogenic media and stained with Alizarin red at 0 and 3 days. D, The Alizarin red staining was extracted and quantified. The wavelength was measured at 562 nm. Data are representative of three independent experiments and values are means ± SEM. ***P < .001, compared with control siRNA‐transfected cells

To further confirm the role of NCAM in hypertrophic chondrocyte differentiation, chondrogenic cell line ATDC5 was used as an alternative in vitro model for chondrocyte differentiation. We developed mixed cells with stable NCAM downregulation using plasmid‐based small interfering RNA (siRNA). The expression of NCAM in the siRNA‐transfected cells was lower compared with that of control cells transfected with scrambled siRNA (Figure 3A). Gene silencing of NCAM increased the expression of RunX2 after a 3‐day chondrogenic induction (Figure 3B). In addition, Alizarin red staining and quantitative analysis of the calcium content in the NCAM‐silenced cells showed increased mineral accumulation as compared to control siRNA‐transfected cells (Figure 3C,D). Together, these data further support our hypothesis that NCAM plays an important role in chondrocyte hypertrophy during chondrogenic differentiation.

### 3.4 ERK signaling contributes to NCAM deficiency‐induced chondrocyte hypertrophy

**Figure 4:**
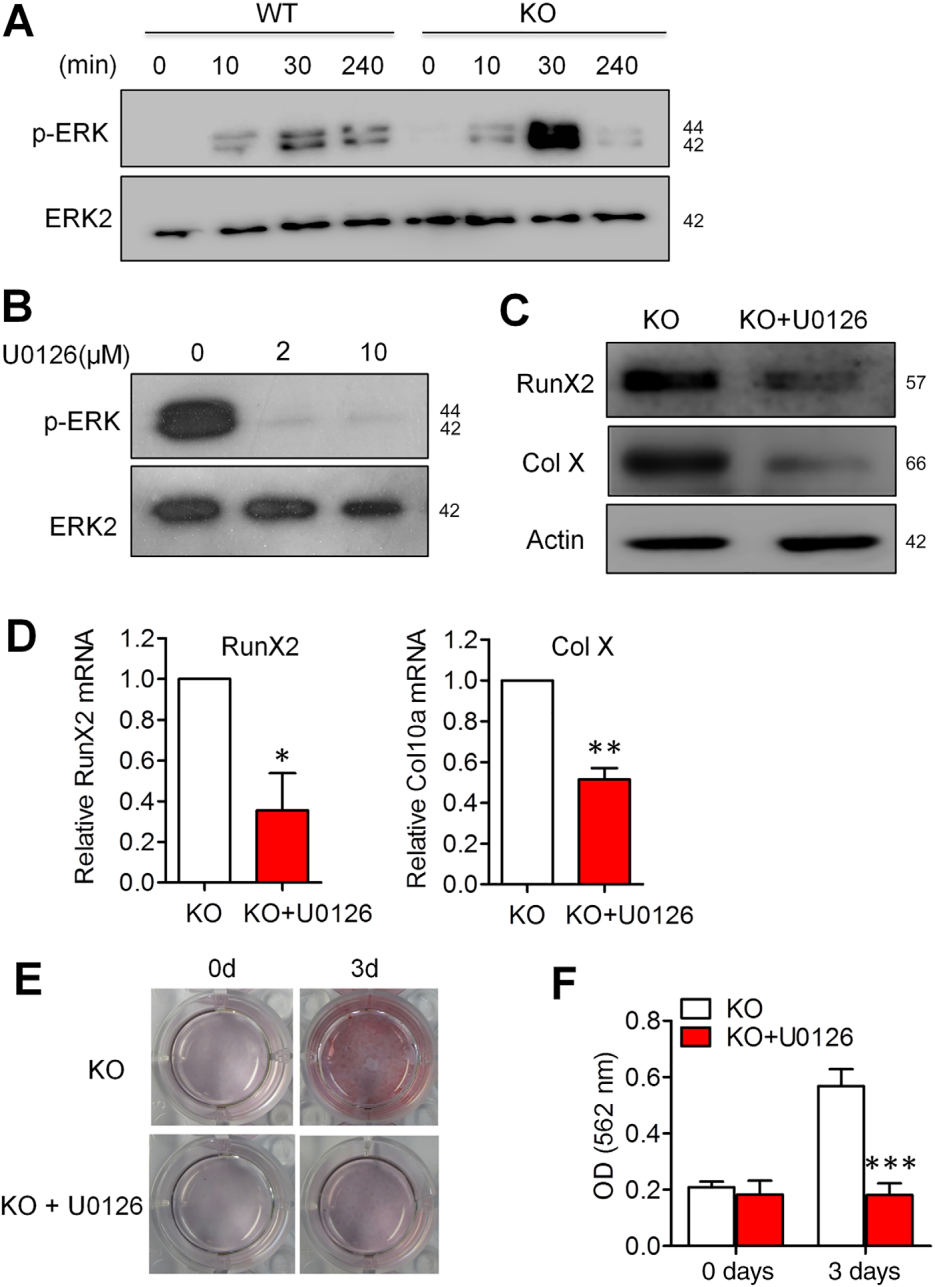
ERK/MAPK signaling contributes to neural cell adhesion molecule (NCAM) deficiency‐induced hypertrophic chondrocyte differentiation. A, Wild‐type (WT) and *Ncam* ^−/−^ (KO) mesenchymal stem cells (MSCs) were treated with differentiation media for 0, 5, 30, and 240 minutes and the phosphorylated ERK1/2 were analyzed by immunoblotting. Total ERK2 was detected as loading controls. B, *Ncam* ^−/−^ MSCs were pretreated with the MEK inhibitor U0126 (0, 2, or 10 μM) for 1 hour prior to chondrogenic induction for 30 minutes. Phosphorylated ERK was then analyzed. C, D, In the presence of U0126 (10 μM), cells were induced with differentiation medium for 3 days and the expression of RunX2 and Col X were examined by immunoblotting (C) and real‐time PCR (D). The results are expressed as the mean ± SEM of three independent experiments. *P < .05, **P < .01, compared with *Ncam* ^−/−^ MSCs without U0126 treatment. E, In the presence of U0126 (10 μM), cells were induced with chondrogenic media for 3 days and stained with Alizarin red. F, The Alizarin red staining was extracted and measured at 562 nm (n = 3; mean ± SEM; *P < .05 vs *Ncam* ^−/−^ MSCs without U0126 treatment)

It has been shown that the ERK signaling pathway plays an essential role in hypertrophic and terminal differentiation events of growth plate chondrocytes.^28^ The activation of ERK signaling was examined to explore the mechanism underlying NCAM deficiency induced hypertrophic chondrocyte differentiation. As shown in Figure 4A, a stronger phosphorylation of ERK was observed in chondrocyte‐differentiated *Ncam* ^−/−^ MSCs as compared to the WT cells. To determine the significance of ERK activation under these conditions, we applied the ERK inhibitor U0126 during chondrogenic induction. As expected, phosphorylation of ERK was inhibited by U0126 (Figure 4B). As a result of ERK inhibition, the mRNA expression and protein production of Col X and RunX2 in *Ncam* ^−/−^ MSCs were markedly downregulated (Figure 4C,D). Accordingly, matrix mineralization and accumulation of calcium were also inhibited by U0126 (Figure 4E,F) in *Ncam* ^−/−^ MSCs. These data indicate that ERK signaling is involved in NCAM deficiency‐induced chondrocyte hypertrophy.

To further illustrate the contribution of ERK signaling in hypertrophic differentiation, we upregulated ERK activation after chondrogenic induction for 30 minutes in mouse MSCs by transfecting the constitutively active form of MEK (EMEK) (Figure S4A). As shown in Figure S4B, the introduction of EMEK could increase the expression of RunX2 at protein levels. Alizarin red staining and quantitative analysis also revealed that ERK activation leads to the increase in mineral accumulation (Figure S4C,D).

### 3.5 NCAM inhibits chondrocyte hypertrophy in cellular OA model

**Figure 5:**
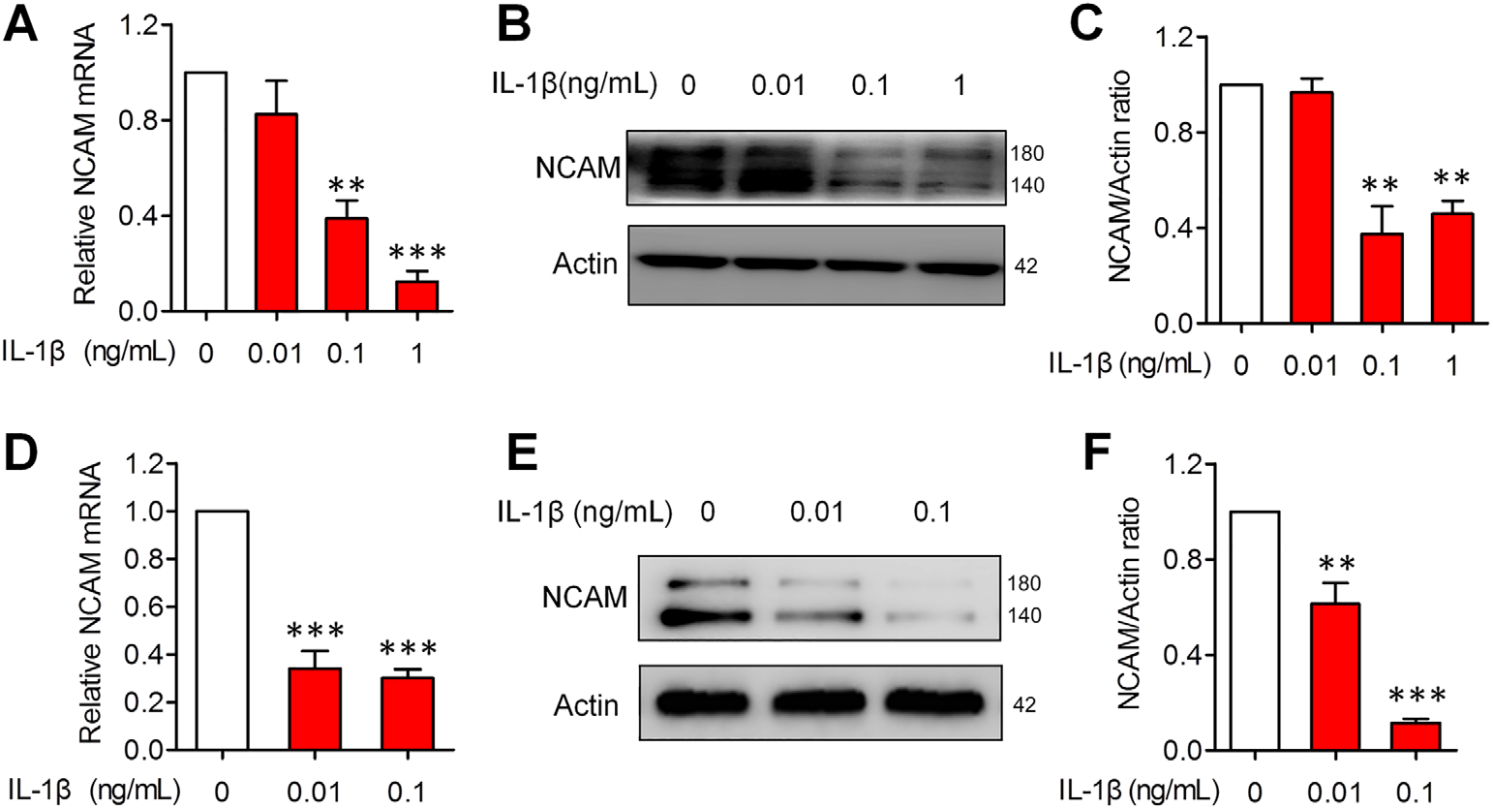
Neural cell adhesion molecule (NCAM) expression is downregulated in IL‐1β‐stimulated mesenchymal stem cells (MSCs) and ATDC5 cells. A, B, MSCs were induced with or without IL‐1β (0.01, 0.1, and 1 ng/mL) for 1 hour and then underwent chondrogenic induction for 3 days. A, The mRNA levels of NCAM were determined by qPCR, the results are expressed as the mean ± SEM of three independent experiments. **P < .01, ***P < .001, compared with cells without IL‐1β treatment. B, The protein levels of NCAM were determined by Western blotting. C, Level of NCAM was quantified by densitometry and normalized to β‐actin (n = 3; mean ± SEM; **P < .01, compared with cells without IL‐1β treatment). D, E, ATDC5 cells were stimulated with or without IL‐1β (0.01 and 0.1 ng/mL) for 1 hour and then induced with differentiation medium for 3 days. D, The mRNA levels of NCAM were determined by qPCR (n = 3; mean ± SEM; ***P < .001, compared with cells without IL‐1β treatment). E, The protein levels of NCAM were determined by Western blotting. F, Level of NCAM was quantified by densitometry and normalized to β‐actin. Data are representative of three independent experiments and values are means ± SEM. **P < .01, ***P < .001, compared with cells without IL‐1β treatment

It has been reported that the level of interleukin (IL)‐1β is elevated in the synovial fluid, subchondral bone, and cartilage of joints in OA patients.^29^, ^30^ Research has shown that IL‐1β induces calcification and increases the expression of hypertrophic gene MMP‐13 and Col X.^31^ We showed here that IL‐1β upregulates the expression of hypertrophic makers RunX2 and Col X in mouse MSCs (Figure S5A). These data suggested that IL‐1β stimulation was a useful cellular OA model in vitro. In such model of OA, we found that the expression of NCAM was significantly reduced at the mRNA and protein level in mouse MSCs induced by IL‐1β (Figure 5A‐C). In addition, we examined NCAM expression in chondrogenic cells; the level of NCAM was lower in IL‐1β‐stimulated ATDC‐5 cells (Figure 5D‐F), with increased expression of hypertrophic maker RunX2 (Figure S5B). In contrast to MSCs, ATDC‐5 cells were more sensitive to IL‐1β stimulation for NCAM downregulation and chondrocyte hypertrophy, even at a lower concentration (0.01 ng/mL). To further confirm the reduced expression of NCAM in experimental OA, MIA‐induced OA model was established, as demonstrated by the results of H&E staining and ELISA detection of inflammatory cytokines (Figure S6A,B). Western blotting analysis showed that NCAM expression was downregulated in MIA‐induced OA in rats (Figure S6C,D).

**Figure 6:**
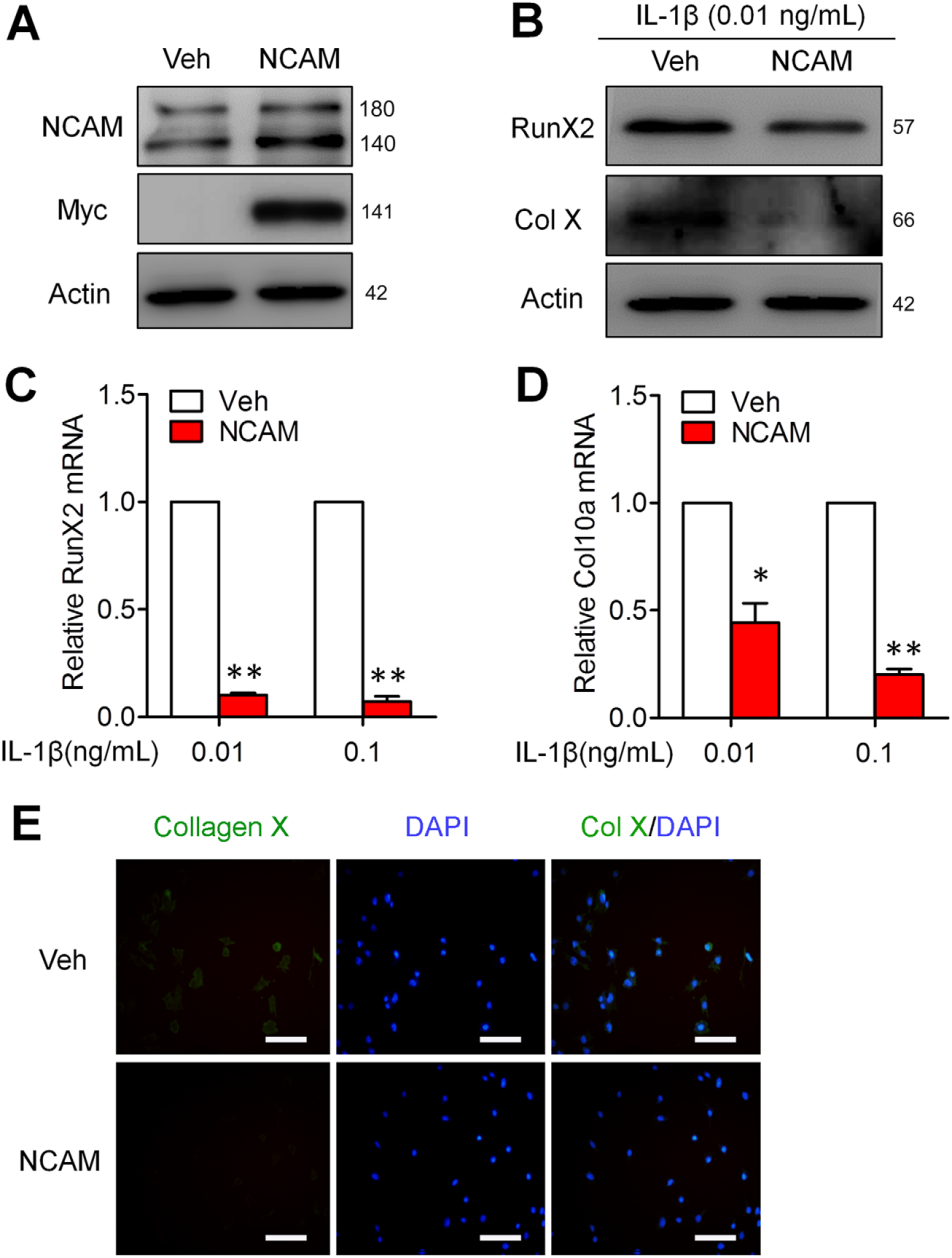
Overexpression of neural cell adhesion molecule (NCAM) inhibits hypertrophic chondrocyte differentiation in IL‐1β‐stimulated ATDC5 cells. A, Cells were stably transfected with pcDNA4/Myc‐NCAM or pcDNA4/Myc (Veh). The expression of NCAM was analyzed by immunoblotting with antibodies against NCAM or c‐Myc tag, respectively. B, Cells were treated with chondrocyte‐differentiation medium for 3 days in the presence of IL‐1β (0.01 ng/mL) and the expressions of RunX2 and Col X were detected by immunoblotting. C, D, cells were differentiated for 3 days with 0.01 and 0.1 ng/mL IL‐1β, and qPCR was performed to examine the mRNA levels of RunX2 (C) and Col X (D). (n = 3; mean ± SEM; *P < .05, **P < .01, compared with Veh group). E, The expression of Col X in differentiated Veh and NCAM‐overexpressing cells with IL‐1β (0.01 ng/mL) was detected by immunofluorescence microscopy (original magnification ×200, scale bars 100 μm)

To confirm the function of NCAM in OA model, chondrogenic cells were transfected with plasmids expressing full‐length mouse NCAM. Stable NCAM‐overexpressing cell lines were obtained by selection with neomycin (Figure 6A) and then submitted to chondrogenic differentiation in the presence of IL‐1β. NCAM‐overexpressing cells showed significant decreases in hypertrophic markers RunX2 and Col X, at the protein (Figure 6B) and mRNA (Figure 6C,D) levels as compared with control cells transfected with empty vector. Immunofluorescence results also showed a lower fluorescence intensity of Col X in NCAM‐overexpressing cells than vector control ones (Figure 6E). These results suggest that NCAM may play a vital role in chondrocyte hypertrophy of OA in vitro.

## 4 DISCUSSION

The process of chondrogenesis includes mesenchymal cell recruitment and condensation, chondrocyte differentiation, and maturation. Chondrocytes mature to two different fates. They either remain as chondrocytes, ceasing differentiation, and forming persistent cartilage located on the surface of the joint, or differentiate into hypertrophic chondrocytes contributing to the mineralization of ECM and formation of the growth plate.^32^, ^33^ In the present study, we provide the first evidence that NCAM plays a novel role in chondrocyte hypertrophy of chondrogenic differentiation. NCAM regulates chondrocyte hypertrophy in experimental OA in vitro and in vivo. Moreover, NCAM deficiency enhances hypertrophic chondrocyte differentiation in both MSCs and chondrogenic cells, in which ERK activation plays an important role.

The differentiation phase of MSCs chondrogenesis can be divided into distinct stages, but there are no critical spatiotemporal cues in chondrogenic differentiation in vitro. Chondrogenesis of MSCs is induced in vitro with defined media containing TGF‐β where chondrocyte differentiation genes Sox9 and collagen II are upregulated. Meanwhile, the hypertrophic markers including Col X and RunX2 are also expressed continuously.^8^ During normal chondrogenesis in vitro, both chondrogenic genes and hypertrophic markers are concomitantly expressed in equilibrium.^34^, ^35^ NCAM promotes precartilaginous condensation, but it is not required for initiation of chondrocyte differentiation.^19^, ^23^ In this study, we also showed that NCAM deficiency did not affect the chondrocyte differentiation step because there was no significant difference in Sox9 and collagen II expression (Figure S3) and aggrecan accumulation^36^ between WT and *Ncam* ^−/−^ MSCs. Interestingly, data in our present study demonstrated that the hypertrophic differentiation in *Ncam* ^−/−^ MSCs is enhanced as demonstrated by increased induction of RunX2 and Col X, and increased mineral accumulation. NCAM silencing also promoted the differentiation of chondrocyte hypertrophy in the chondrogenic cell line ATDC5. These findings indicate that NCAM plays a crucial role in chondrocyte hypertrophy of chondrogenic differentiation in vitro. NCAM deficiency enhances the hypertrophic differentiation and may disrupt the benign balance between expression of chondrogenic genes and expression of hypertrophic markers, which may result in abnormal chondrogenesis dominated by chondrocyte hypertrophy.

Multiple signaling molecules were shown to regulate the differentiation of chondrocytes from the initial induction of mesenchymal progenitor cells to the terminal maturation of hypertrophic chondrocytes,^9^, ^11^ including mitogen‐activated protein kinase (MAPK) pathways.^37^, ^38^ The role of ERK/MAPK pathway remains puzzling that some studies report a positive, and others a negative, action on chondrocyte proliferation and hypertrophy.^39^ In our study, we demonstrated that the activation of ERK signaling was increased in *Ncam* ^−/−^ MSCs. We also showed that hypertrophic markers and mineral accumulation were blocked by ERK inhibitor U0126. In contrast, the constitutively active form of ERK (EMEK) could increase chondrocyte hypertrophy in chondrogenic differentiation of MSCs. The regulation of the ERK pathway occurs mainly through GTPase Ras, which recruits the Raf family and activates MEK1 and MEK2.^39^ A previous study demonstrated that c‐Raf, MEK1/2, and ERK1/2 are required for the expression of Col X.^40^ ERK1/2 is also identified as a regulator of the expression of skeletal development gene Runx2.^41^ ERK activation was detected primarily in hypertrophic chondrocytes and a MEK inhibitor coincided with a delay in chondrocyte maturation.^42^ A recent study demonstrated that phosphorylation of ERK is overexpressed in human OA cartilage and subchondral bone tissue, and ERK inhibitor U0126 can inhibit the hypertrophic changes in OA articular cartilage chondrocytes.^43^ These results are consistent with our data that NCAM deficiency elevated chondrocyte hypertrophy by ERK activation, supporting a positive role of ERK signaling in OA cartilage hypertrophic changes. In our previous work, ERK is found to be activated by NCAM and the activation of ERK is partially responsible for the migration of MSCs.^36^ However, the present study showed a negative regulation of NCAM on ERK in the chondrocyte hypertrophy in chondrogenic differentiation. The results are very interesting and we are curious why NCAM behaves so differently in cell migration and chondrogenic differentiation of MSCs, which needs to be further investigated.

OA is characterized by inflammation and catabolism in joint environment, leading to progressive degeneration of cartilage.^5^ In the present study, we found that expression of NCAM was decreased in both MSCs and chondrogenic cells stimulated with IL‐1β, an inflammatory cytokine frequently used in developing an OA cellular model. Chondrocyte hypertrophy‐like changes, such as hypertrophy genes expression and cartilage calcification, are reported in (experimental) OA.^44^ In our study, the features of excessively hypertrophic chondrocyte differentiation induced by NCAM deficiency are similar to those of hypertrophy‐like chondrocytes in OA. Adhesion of adhesion molecules such as N‐cadherin and NCAM are essential for differentiation of mesenchymal cells into chondrocytes.^32^ Here, the role of NCAM was determined in WT and *Ncam* ^−/−^ OA mice. Our data imply that NCAM can also be considered as a potential regulator of chondrocyte hypertrophy in the pathogenesis of OA. In recent years, a great deal of attention has been focused on cell‐based therapeutic strategy for cartilage degeneration using MSCs,^45^ however, chondrogenic differentiation of MSCs is usually inefficient that excessive chondrocyte hypertrophy is observed under inflammatory intra‐articular conditions caused by OA. How to inhibit chondrocyte hypertrophy‐like changes in treatment of cartilage damage using MSCs remains a challenge.^46^, ^47^ In this study, we demonstrated that overexpression of NCAM prevents hypertrophic differentiation of chondrogenic cells in the inflammatory environment induced by IL‐1β, suggesting NCAM as a novel target for development of more efficient approach to repair cartilage injury by inhibition of chondrocyte hypertrophy.

In summary, we demonstrate here that NCAM deficiency promotes chondrocyte hypertrophy in chondrogenic differentiation of MSCs. NCAM is downregulated in cellular OA model and regulates chondrocyte hypertrophy in experimental OA in vitro and in vivo. The results support NCAM as a new regulator of chondrocyte hypertrophy in chondrocyte differentiation and OA pathogenesis. Our findings provide potential novel strategies for the cartilage repair of OA treatment.

## CONFLICT OF INTEREST

The authors indicated no potential conflicts of interest.

## AUTHOR CONTRIBUTIONS

B.‐F.C., Z.‐W.F.: conception and design, final approval of manuscript, financial support, manuscript writing; J.J.L.: collection and/or assembly of data, data analysis and interpretation; H.‐J.Y.: financial support, administrative support; L.W.: data analysis and interpretation; H.‐H.Y.: manuscript writing, collection and/or assembly of data; J.‐J.B.: financial support, data analysis and interpretation; Y.‐X.G.: collection and/or assembly of data; S.‐J.C., M.W.: data analysis and interpretation.

## Supporting information


**Figure S1** MIA‐induced OA was established in mice. (**A**) Safranin O staining of articular cartilage sections (n = 5; original magnification ×200; Scale bars = 50 μm). (**B**) Plasma was obtained and the levels of IL‐1β and TNF‐α were measured by ELISA. The results are expressed as the mean ± SD. *P < 0.05, **P < 0.01, compared with the normal saline (NS) group.

**Figure S2** Wild‐type (WT) and *Ncam* ^*−/−*^ (KO) MSCs were chondrocyte‐differentiated and stained with Alcian blue or Alizarin red. (**A**) WT cells were induced with chondrogenic media for 0, 1, 3, 7 days and 10 days, and stained with Alcian blue and Alizarin red, respectively. (**B**) chondrocyte‐differentiated WT and KO cells were stainied by Alizarin red (original magnification ×200). (**C**) WT and KO cells were chondrocyte‐differentiated and stained with Alcian blue.

**Figure S3** The mRNA expression of Sox9 (**A**) and ColII(**B**) in chondrocyte‐differentiated wild‐type (WT) and *Ncam* ^*−/−*^ (KO) MSCs was analysed by real‐time PCR. The results are expressed as the mean ± SEM of three independent experiments. *P < 0.05 and **P < 0.01, compared with differentiated WT MSCs.

**Figure S4** Activation of ERK/MAPK signaling boosts hypertrophic chondrocyte differentiation in MSCs. (**A**) WT cells were stably transfected with constitutively active form of MEK (EMEK) or vehicle plasmid (Veh). The level of phosphorylated ERK was analysed by immunoblotting. Total ERK2 served as a loading control. (**B**) Cells transfected with Veh or EMEK were subjected to chondrocyte differentiation for 3 days, and the expression of RunX2 was analyzed by immunoblotting. (**C**) Cells were chondrocyte‐differentiated for 5 days and stained with Alizarin red. (**D**) The Alizarin red staining was extracted and quantified at a wavelength of 562 nm. Data are representative of three independent experiments and values are means ± SEM. ***P < 0.001, compared with Veh group.

**Figure S5** IL‐1β induces chondrocyte hypertrophy in MSCs and ATDC‐5 cells. (**A**) MSCs were stimulated with IL‐1β (0.01, 0.1 and 1 ng/mL) for 1 hour and then induced with chondrogenic differentiation media for 3 days. The expressions of RunX2 and Col X were analysed by Western blotting. β‐actin served as a loading control. (**B**) ATDC‐5 cells were induced with IL‐1β (0.01, 0.1 and 1 ng/mL) for 1 hour and then underwent chondrogenic induction for 3 days. The expression of RunX2 was analysed by Western blotting. β‐actin served as a loading control.

**Figure S6** NCAM expression is downregulated in MIA‐induced OA rats. (**A**) HE staining of rat joints sections (n = 7; original magnification ×100; Scale bars = 100 μm). The normal saline (NS) was used as sham group. (**B**) Plasma was obtained and the levels of TNF‐α and IL‐1β were measured by ELISA. The results are expressed as the mean ± SEM. *P < 0.05 or **P < 0.01, compared with the NS group. (**C**) NCAM level in cartilage tissue was examined by immunoblotting. β‐actin served as a loading control. (**D**) Level of NCAM was quantified by densitometry and normalized to β‐actin. Data are representative of three independent experiments and values are means ± SD. *P < 0.05, compared with the NS group.

**Table S1** Primers for real‐time PCR.

## Data Availability

The data that support the findings of this study are available from the corresponding author upon reasonable request.
