# Supplementary figures and images for: Neural cell adhesion molecule regulates chondrocyte hypertrophy in chondrogenic differentiation and experimental osteoarthritis
Source: Stem Cells Transl Med. 2019 Nov 19;9(2):273–83. doi: 10.1002/sctm.19-0190 (PMC6988767; doi:10.1002/sctm.19-0190)

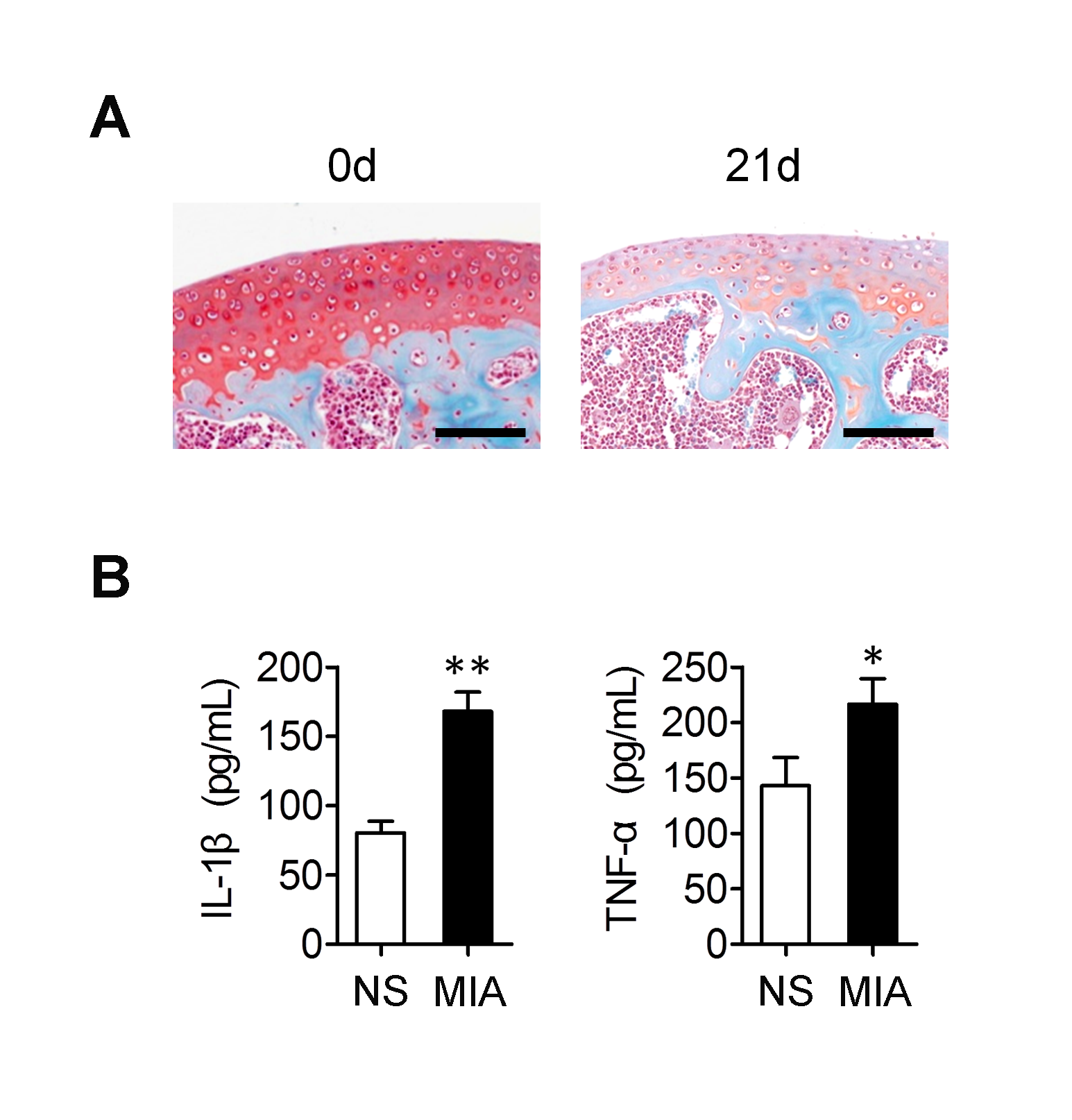

Supplement: Supplementary file 1 — Figure S1 MIA‐induced OA was established in mice. (A) Safranin O staining of articular cartilage sections (n = 5; original magnification ×200; Scale bars = 50 μm). (B) Plasma was obtained and the levels of IL‐1β and TNF‐α were measured by ELISA. The results are expressed as the mean ± SD. *P < 0.05, **P < 0.01, compared with the normal saline (NS) group. [file SCT3-9-273-s001.tif]

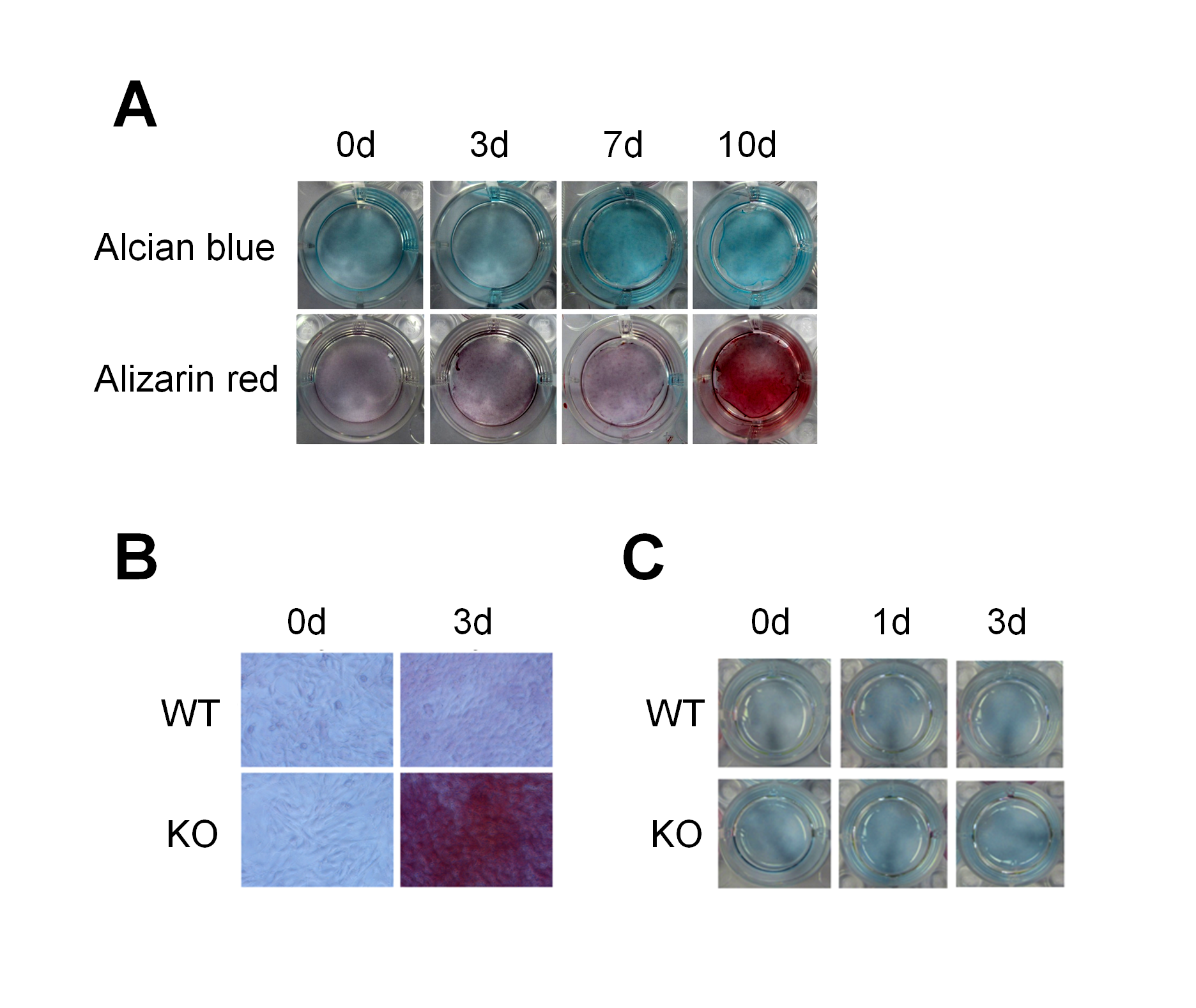

Supplement: Supplementary file 2 — Figure S2 Wild‐type (WT) and Ncam −/− (KO) MSCs were chondrocyte‐differentiated and stained with Alcian blue or Alizarin red. (A) WT cells were induced with chondrogenic media for 0, 1, 3, 7 days and 10 days, and stained with Alcian blue and Alizarin red, respectively. (B) chondrocyte‐differentiated WT and KO cells were stainied by Alizarin red (original magnification ×200). (C) WT and KO cells were chondrocyte‐differentiated and stained with Alcian blue. [file SCT3-9-273-s002.tif]

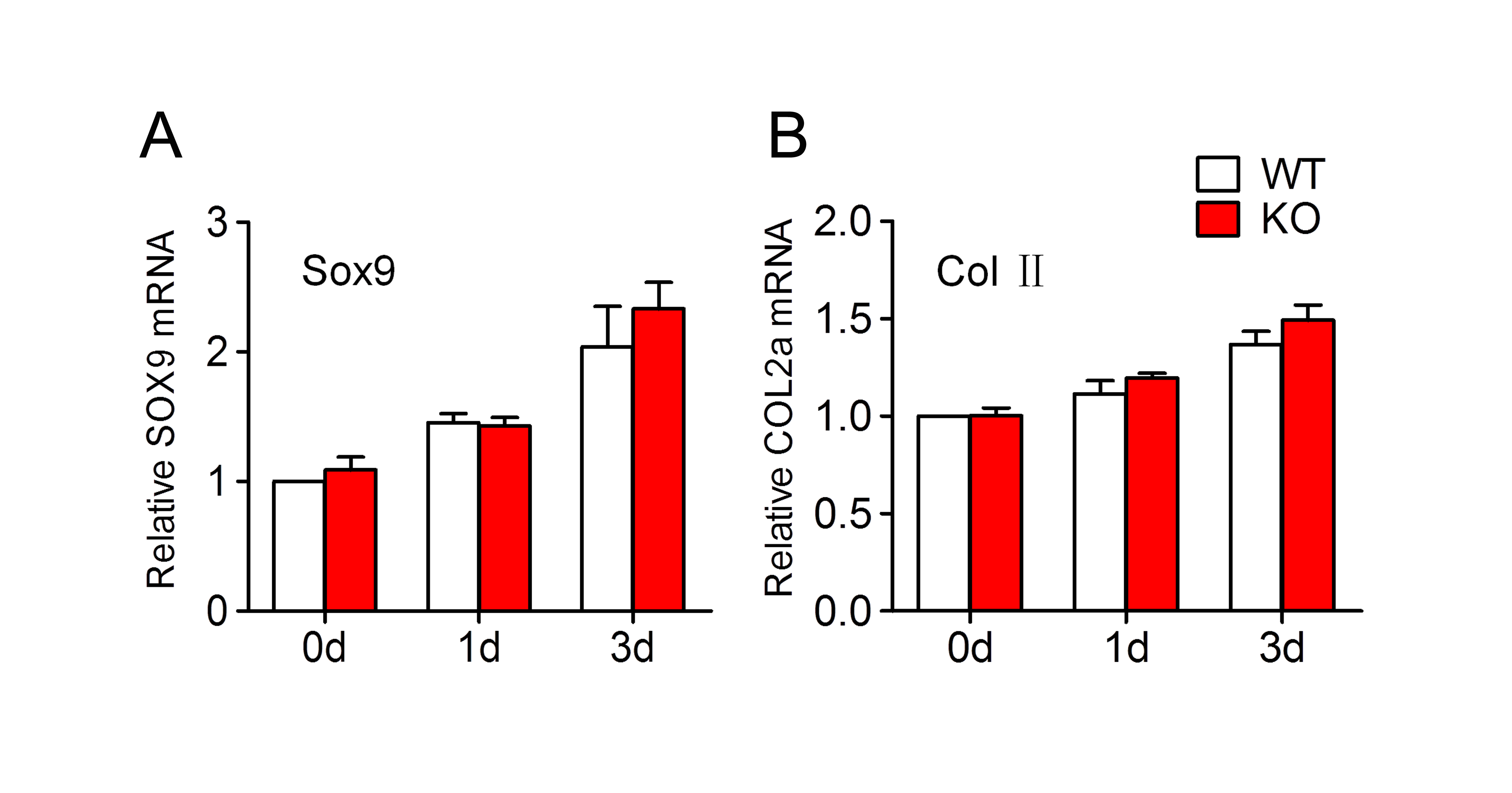

Supplement: Supplementary file 3 — Figure S3 The mRNA expression of Sox9 (A) and ColII(B) in chondrocyte‐differentiated wild‐type (WT) and Ncam −/− (KO) MSCs was analysed by real‐time PCR. The results are expressed as the mean ± SEM of three independent experiments. *P < 0.05 and **P < 0.01, compared with differentiated WT MSCs. [file SCT3-9-273-s003.tif]

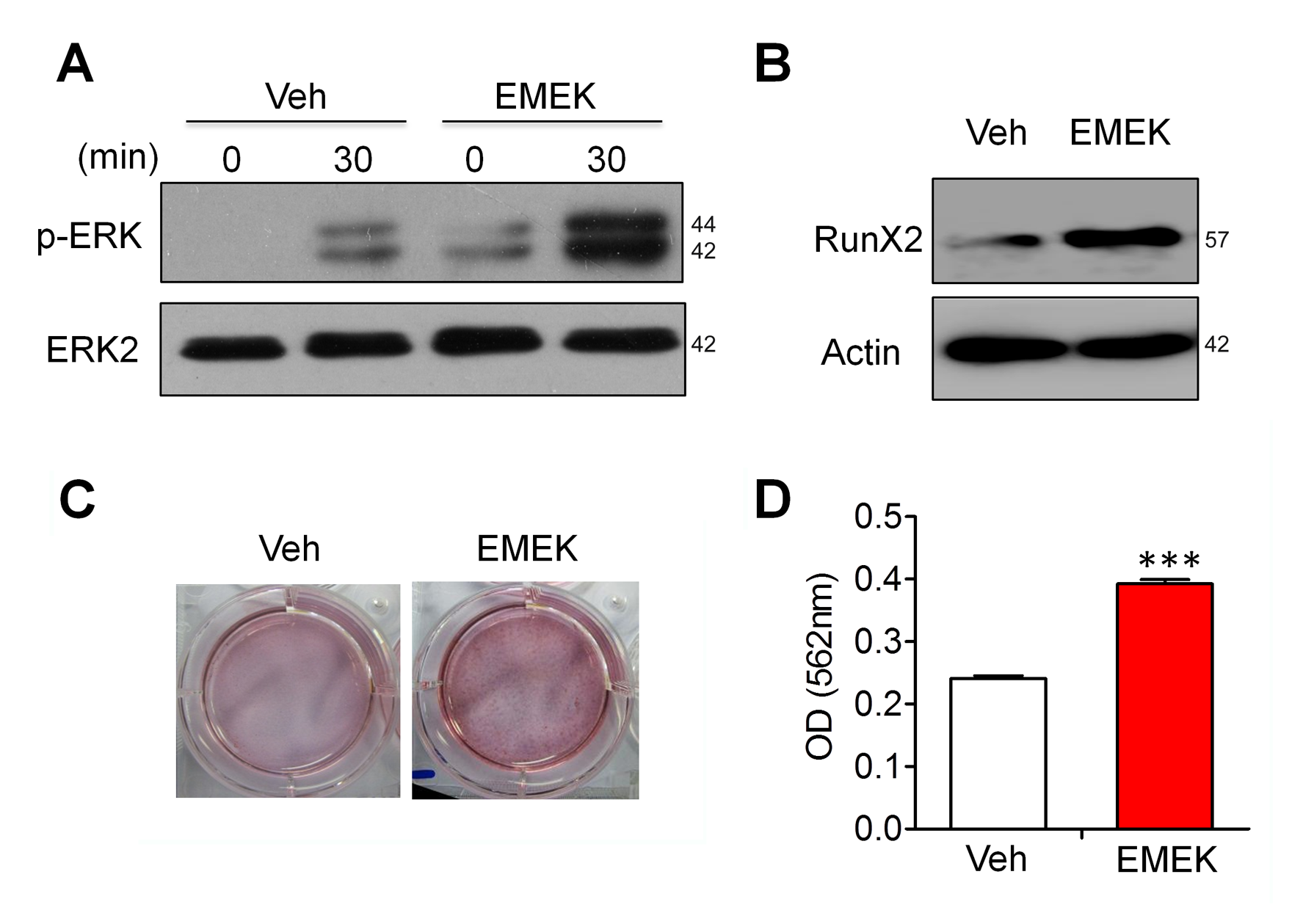

Supplement: Supplementary file 4 — Figure S4 Activation of ERK/MAPK signaling boosts hypertrophic chondrocyte differentiation in MSCs. (A) WT cells were stably transfected with constitutively active form of MEK (EMEK) or vehicle plasmid (Veh). The level of phosphorylated ERK was analysed by immunoblotting. Total ERK2 served as a loading control. (B) Cells transfected with Veh or EMEK were subjected to chondrocyte differentiation for 3 days, and the expression of RunX2 was analyzed by immunoblotting. (C) Cells were chondrocyte‐differentiated for 5 days and stained with Alizarin red. (D) The Alizarin red staining was extracted and quantified at a wavelength of 562 nm. Data are representative of three independent experiments and values are means ± SEM. ***P < 0.001, compared with Veh group. [file SCT3-9-273-s004.tif]

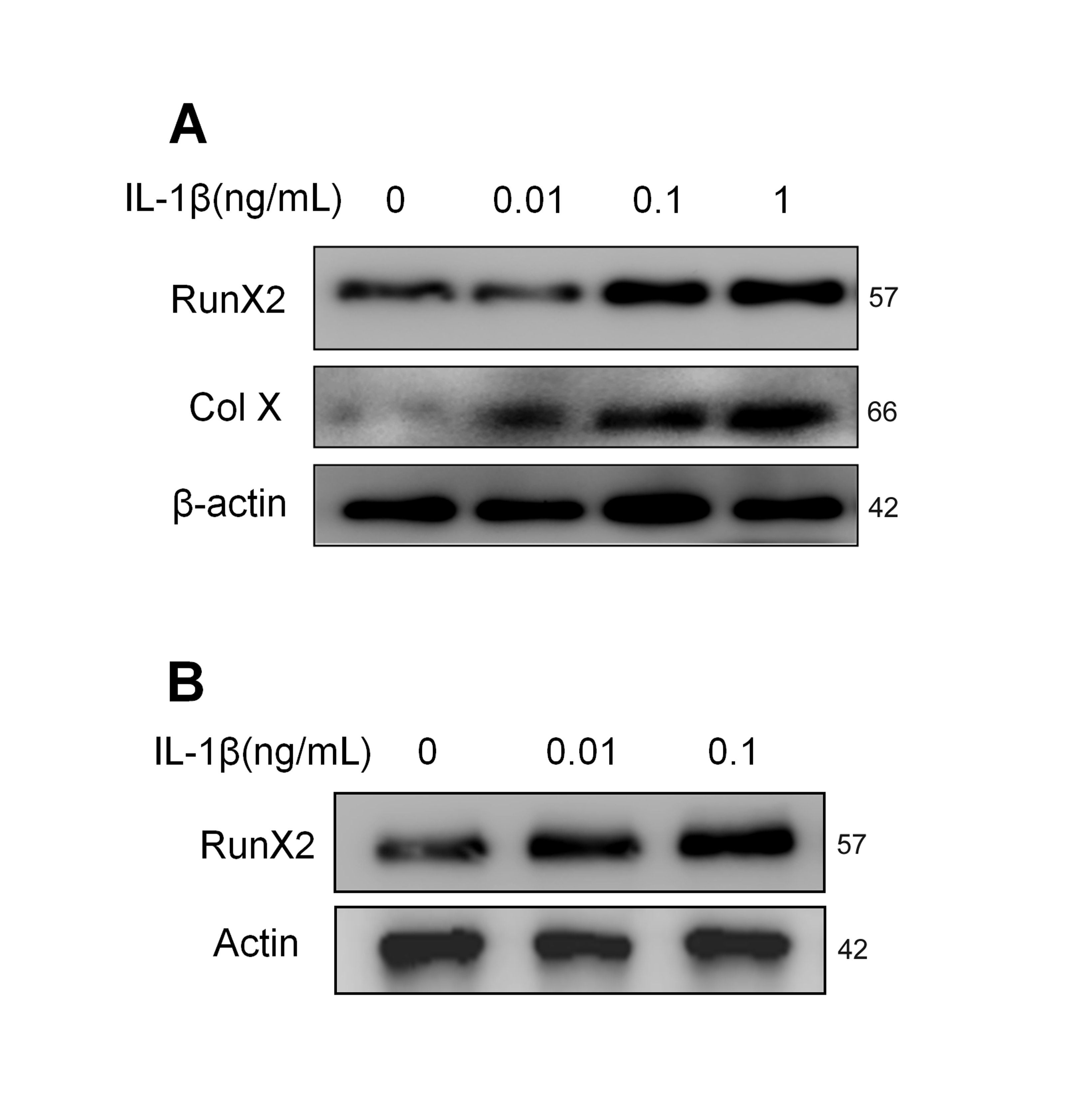

Supplement: Supplementary file 5 — Figure S5 IL‐1β induces chondrocyte hypertrophy in MSCs and ATDC‐5 cells. (A) MSCs were stimulated with IL‐1β (0.01, 0.1 and 1 ng/mL) for 1 hour and then induced with chondrogenic differentiation media for 3 days. The expressions of RunX2 and Col X were analysed by Western blotting. β‐actin served as a loading control. (B) ATDC‐5 cells were induced with IL‐1β (0.01, 0.1 and 1 ng/mL) for 1 hour and then underwent chondrogenic induction for 3 days. The expression of RunX2 was analysed by Western blotting. β‐actin served as a loading control. [file SCT3-9-273-s005.tif]

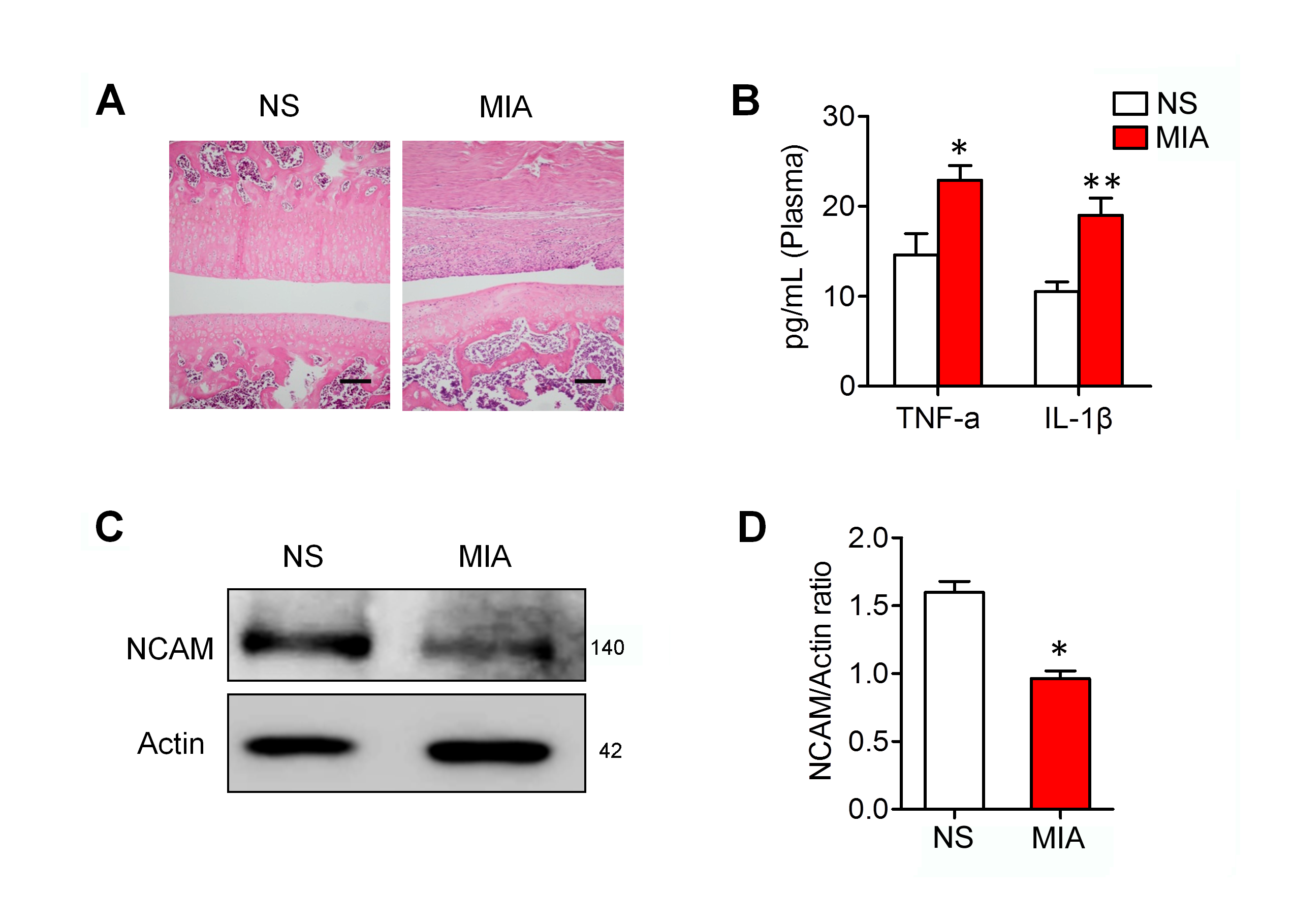

Supplement: Supplementary file 6 — Figure S6 NCAM expression is downregulated in MIA‐induced OA rats. (A) HE staining of rat joints sections (n = 7; original magnification ×100; Scale bars = 100 μm). The normal saline (NS) was used as sham group. (B) Plasma was obtained and the levels of TNF‐α and IL‐1β were measured by ELISA. The results are expressed as the mean ± SEM. *P < 0.05 or **P < 0.01, compared with the NS group. (C) NCAM level in cartilage tissue was examined by immunoblotting. β‐actin served as a loading control. (D) Level of NCAM was quantified by densitometry and normalized to β‐actin. Data are representative of three independent experiments and values are means ± SD. *P < 0.05, compared with the NS group. [file SCT3-9-273-s006.tif]
