# Supplementary material for: Neural cell adhesion molecule regulates chondrocyte hypertrophy in chondrogenic differentiation and experimental osteoarthritis
Source: Stem Cells Transl Med. 2019 Nov 19;9(2):273–83. doi: 10.1002/sctm.19-0190 (PMC6988767; doi:10.1002/sctm.19-0190)
Supplement: Supplementary file 7 — Table S1 Primers for real‐time PCR. [file SCT3-9-273-s007.doc]

**Table S1** Primers for real-time PCR.

| **Gene** | **Primer** | **Sequence (5'-3')** |
| --- | --- | --- |
| NCAM | Forward | TCTACCCTCACCATCTACAACG |
|  | Reverse | GCTGACCACATCACAGACAATC |
| RunX2 | Forward | CGAAATGCCTCCGCTGT TAT |
|  | Reverse | TGAGGAATGCGCCCTAAATC |
| Col 10α | Forward | CTGCTAATGTTCTTGACCCTGGTT |
|  | Reverse | GGAATGCCTTGTTCTCCTCTTACTG |
| Sox9 | Forward | GAAGAAGGAGAGCGAGGAAGATA |
|  | Reverse | TTGTGCAGATGCGGGTACTG |
| Col 2α | Forward | AGCAGAGGTGATCGTGGTGACAAG |
|  | Reverse | TCATACCCTCCAGCCATCTGA |
| GAPDH | Forward | CTTCAACAGCAACTCCCACT |
|  | Reverse | GTCCAGGGTTTCTTACTCCT |
